# Embracing complexity at the physiology and behaviour interface will benefit conservation science

**DOI:** 10.1093/conphys/coag021

**Published:** 2026-04-08

**Authors:** Suzanne Currie, M Danielle McDonald, Katherine A Sloman

**Affiliations:** Department of Biology, University of British Columbia, Okanagan Campus, Kelowna, BC V1V 1V7, Canada; Rosenstiel School of Marine, Atmospheric, and Earth Science, 4600 Rickenbacker Causeway, University of Miami, Miami, FL 33149-1098, USA; School of Health and Life Sciences, Lanarkshire Campus - Stephenson Place, University of the West of Scotland, Paisley G72 0LH, Scotland

**Keywords:** Abiotic, adaptation, biotic, developmental stage, environmental challenge, hypoxia, physiology/behaviour interface, reproductive status, social context, temperature, thermal preference

## Abstract

Over recent years, the opinion that physiology or behaviour are the most sensitive indicators of environmental change has become less prominent, with the recognition that complex dynamic feedback loops exist between an individual’s physiology and behaviour. The fluidity of the physiology/behaviour interface and its sensitivity to abiotic factors, such as exposure to temperature change and low oxygen (hypoxia), or biotic factors, such as genetics, reproductive status or social interaction, form an organism’s context. Individual contexts can make the way animals respond to an environmental challenge difficult to predict and conservation efforts incredibly challenging. Our Perspective draws on examples from across the animal kingdom presented at the 2024 Society for Experimental Biology symposium, ‘Linking Physiology and Behaviour in a Changing World’, which investigated the interplay between an animal’s context and the environmental challenges they experience, in shaping the physiology/behaviour interface. Our *Perspective* highlights that if we want to address the conservation and biodiversity implications of the rapid environmental change we now face, it is critical that we continue to move away from reductionist methodologies and adopt holistic interdisciplinary approaches to provide conservation biologists with the tools they need to solve our most pressing conservation challenges.

## Introduction

One of the biggest challenges facing conservation scientists is how to predict the response of an organism to a rapidly changing world. Historically, scientists have considered the physiological and behavioural responses to environmental change as separate entities (Reese, 1996; Debaere *et al.*, 2024). However, over the past 20 years, there has been increasing attention given not only to the inextricable links that exist between physiology and behaviour, but also to their importance in understanding how animals cope with environmental change (Gilmour *et al.*, 2005; Killen *et al.*, 2013). With the emergence of conservation physiology (Cooke *et al.*, 2013), the scientific discipline applying physiological tools and concepts to understand and solve conservation challenges, there has been strong advocacy for the integration of animal behaviour and physiology (Cooke *et al.*, 2014). Traditionally, using hierarchical levels of biological organization as a framework for understanding the different ways an organism may respond to a changing/challenging environment (e.g. Fig. 1 in Cooke *et al.*, 2014), physiological processes at the cellular, organ and whole animal level have been considered to drive phenotypes (including behaviours), potentially leading to changes at the population level. With recent progress on the physiology/behaviour interface, it has become increasingly evident that rather than unidirectional causality between physiological processes and animal behaviour, there are fluid, correlative relationships between physiological and behavioural traits under changing environmental conditions (Killen *et al.*, 2013). A complex dynamic feedback loop exists between behaviour and physiology that can make environmental impacts on animals problematic to predict and make conservation efforts and management decisions a moving target.

**Figure 1:**
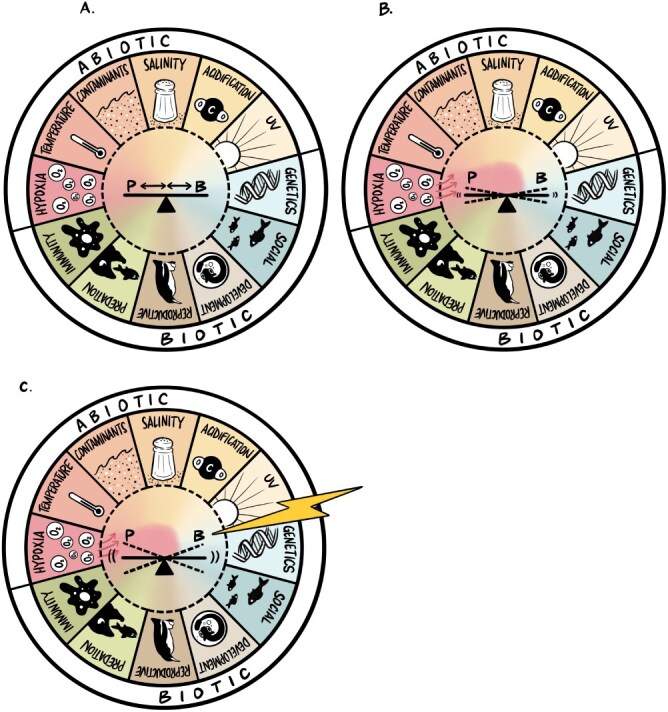
(**A**) The physiology/behaviour interface of an animal is perfused by a variety of abiotic and biotic factors shaping its specific context. Here, the inner circle represents the animal’s context, with the outer coloured ring representing examples of abiotic or biotic factors, which come together to form the context the animal experiences. The physiology/behaviour interface, represented as a seesaw, is a balance where in some contexts behaviour may have a greater influence on physiology, or *vice versa*. The perpetual motion illustrated by the seesaw may sometimes be slow and steady but can also be changed by movement along the seesaw axis as indicated by the arrows. (**B**) The intertwining effects of abiotic (e.g. hypoxia, temperature) and biotic (e.g. sociality, reproduction, predation) factors forming an animal’s context dynamically shift on a continuous basis. The most pervasive factors (hypoxia in B panel of this figure) will change throughout an animal’s life span, and these shifts in influencing factors will affect the physiology/behaviour interface. (**C**) An external (abiotic or biotic) environmental challenge, illustrated here as a lightning bolt, can affect the physiology/behaviour interface directly or may interact with an animal’s existing context influencing how animals respond to the same external challenge.

It can be difficult to capture diagrammatically the complex interactions that represent the physiology/behaviour interface; previous discussions have tended towards flow diagrams and feedback loops. In this *Perspective*, we see the physiology/behaviour interface as a seesaw (Fig. 1A), illustrating the perpetual motion that exists between them. At times, the relative importance of ‘physiology’ or ‘behaviour’ in how an organism responds to environmental change may oscillate, more akin to a feedback loop where one influences the other, and this in turn is reciprocated. Here, one might imagine two children on a seesaw, well balanced in mass, moving continuously, and influencing each other in a relatively slow but steady manner. At other times, the interaction between behaviour and physiology may be so fast and integrated that they cannot be disentangled, better represented by two children moving towards each other in the centre of the seesaw. Thus, the interface represents a perpetual interaction. Recently, Debaere *et al.* (2024) defined physiology as the study of how an organism functions, including the relationship and interactions between numerous mechanisms and processes that operate in living organisms. They argued that behaviour should be considered a physiological process, where they defined physiological processes as coordinated actions and interactions of cells, tissues and organs necessary to achieve a desired response. In their *Commentary*, they recognize the challenges with the integration of behaviour as a physiological process, including the avoidance of oversimplification of either field. In our *Perspective*, we consider recognition of the complexity of an individual’s response to environmental challenge to be most important. From a conservation or management perspective, the response of an animal to environmental change will likely continue to be seen as ‘behavioural’ (e.g. activity, migration and foraging, aggression) or ‘physiological’ (e.g. heart rate, metabolic rate and brain activity) for the foreseeable future. Unfortunately, we likely lack time to wait for different disciplines to agree on terminologies.

Beyond the complexity within an individual, understanding how internal and external environmental contexts can shape the physiology/behaviour interface has also increased in the past decade. Here, we consider an animal’s context to be the agglomeration of all the abiotic and biotic factors it experiences daily, i.e. the background that shapes its physiology/behaviour interface (Fig. 1A). During different stages of its life, for example, during reproductive periods or shifts in seasonal temperature, the factors shaping an animal’s context will change in emphasis. It has long been recognized that physiological and behavioural traits vary across ecological contexts (Careau and Garland Jr, 2012) and that environmental context can strengthen, weaken or even dissolve behavioural–physiological linkages (Sih *et al.*, 2004; Biro and Stamps, 2010; Madliger *et al.*, 2018). For example, early research by Koolhaas *et al.* (1999) demonstrated that behavioural and physiological responses to ‘stress’ can be decoupled depending on environmental demands. Our goal here is to underscore the influence of internal and external contexts on the behaviour–physiology interface and to emphasize consideration of this complexity in conservation and management efforts (see below).

Understanding context is important in predicting how additional environmental challenges will affect the physiology/behaviour interface of an animal (Fig. 1B). Within the literature, the terms ‘environmental challenge’ and ‘stressor’ are often used interchangeably, and as above, we recognize that different fields tend towards different terminologies. Within our *Perspective*, we use ‘environmental challenge’ to describe any (usually short-term) challenge that moves an animal from predictive homeostasis to reactive homeostasis and possibly homeostatic failure or overload as defined by the Reactive Scope Model (Romero *et al.*, 2009; Gilmour *et al.*, 2025). Environmental challenges do not occur in isolation, and recent research has highlighted that impacts can combine additively, synergistically or antagonistically, with the concept of ‘multiple stressor’ approaches applied across a range of disciplines (Orr *et al.*, 2020; Simmons *et al.*, 2021; Pirotta *et al.*, 2022). Thus, connectivity at the physiology/behaviour interface is influenced simultaneously by an animal’s context as well as the environmental challenge(s) they experience (Fig. 1C).

Despite the integration of physiology, behaviour and context occurring as a theme in conservation physiology for over a decade (Killen *et al.*, 2013; Cooke *et al.*, 2014; Madliger *et al.*, 2018), recent reviews of the literature (Debaere *et al.*, 2024; Killen *et al.*, 2026) suggest that while this approach has increased in popularity, a widespread shift in thinking has not yet occurred. Clearly, the complexity of an animal’s behaviour, physiology and interactions with its environment cannot be captured through a single lens (Casarrubea, 2024) or *via* easily quantifiable measures. From our *Perspective*, we hope to highlight how different contexts and challenges influence the physiology/behaviour interface, inspired by the 2024 Society for Experimental Biology symposium, ‘Linking Physiology and Behaviour in a Changing World’, which provided us with integrative stories from across the animal kingdom. Through examples below, we show how studies that consider both behaviour and physiology across a range of contexts contribute to our understanding of what is an incredibly complex picture of how organisms respond to environmental change. Looking ahead, we then ask ‘What next?’ for the field and how might we move forward in informing conservation processes through more unified cross-disciplinary approaches.

## Why Context Matters at the Physiology/Behaviour Interface

There are numerous studies that have considered how abiotic or biotic factors affect links between physiology and behaviour in animals. These factors will often shape an individual’s context but may also present as specific environmental challenges. Well-researched abiotic factors include temperature (Abram *et al.*, 2017; Christensen *et al.*, 2021), hypoxia (Carlson and Parsons, 2001; Richards, 2011; Mattiasen *et al.*, 2020), salinity (e.g. Brischoux *et al.*, 2017; Lorrain-Soligon *et al.*, 2022), ocean acidification (Porteus *et al.*, 2018), pollutants (Nie *et al.*, 2022; Bartling *et al.*, 2024) and habitat change (e.g. urbanization; Oliveira *et al.*, 2021). Examples of biotic factors include external influences such as predation risk (Gvoždík and Boukal, 2021; Guigueno *et al.*, 2025), prey availability (e.g. Cothran *et al.*, 2021), parental care/pre-natal condition (e.g. Munch *et al.*, 2018; Bailey *et al.*, 2024) and social interaction or lack thereof (e.g. Xu *et al.*, 2024; Gilmour *et al.*, 2025; Killen *et al.*, 2026). Additional internal biotic factors that are more usually researched in relation to an animal’s context include genetics and epigenetics (e.g. Seebacher and Krause, 2019), sex (e.g. Colominas-Ciuró *et al.*, 2022) and ontogeny (e.g. Haubrock *et al.*, 2020; James *et al.*, 2025). Many of these studies have started to tease apart the complexity of how these factors affect the physiology/behaviour relationship, including the effects of combining several factors (e.g. Sumasgutner *et al.*, 2023). However, there is increasing focus on the role context plays in shaping an animal’s physiological and/or behavioural response to an environmental challenge. In the following paragraphs, we consider examples that were highlighted in the symposium of how this complexity influences the relationship between behaviour and physiology and how it might complicate conservation and management efforts. We provide suggestions of next steps to harness this complexity to benefit conservation science.

## How Context Shapes the Relationship between Physiology and Behaviour

I. Environmental oxygen can impact the physiology/behaviour relationship through both context and environmental challenge. In guppies (*Poecilia reticulata*), behavioural performance is influenced not only by current oxygen conditions, but also by an individual’s prior acclimation history, independent of changes in physiology (Doddema *et al.*, 2025). In this example, two groups of guppies were investigated; one acclimated to constant normoxia and a second to fluctuating hypoxia. Both groups were given an acute hypoxia challenge followed by reoxygenation. In response to the hypoxia challenge, normoxia-acclimated and fluctuating hypoxia-acclimated guppies experienced a similar physiological response—a reduction in metabolic rate. However, fluctuating hypoxia-acclimated guppies showed altered behavioural sensitivity to hypoxia and reoxygenation compared to normoxia-acclimated guppies (Doddema *et al.*, 2025). Specifically, when normoxia-acclimated guppies were exposed to acute hypoxia, they exhibited a lower escape responsiveness compared to when in normoxia. Their escape responsiveness did not recover after reoxygenation (Doddema *et al.*, 2025). In contrast, fluctuating hypoxia-acclimated guppies had an improved escape responsiveness when exposed to acute hypoxia, compared to when in normoxia, that returned to normoxic levels upon reoxygenation (Doddema *et al.*, 2025). This example illustrates a disconnect between metabolic rate, a whole animal physiological measurement often considered to be the amalgamation of all physiological processes, and behaviour, historically considered to be the manifestation of physiology. This illustrates a well-described hurdle for conservation scientists—that exposure history of an organism likely impacts its future sensitivity. Without appropriate interdisciplinary collaboration (e.g. field ecologists with historic oxygen saturation data), known histories of experimental laboratory animals and consideration of both behavioural and physiological endpoints, we might inaccurately estimate the sensitivity of a given organism to an environmental stressor.

II. Developmental stage is a key intrinsic biotic factor shaping context, affecting interactions of behavioural and physiological processes across different scales of biological organization. This is particularly evident in many ectothermic embryos that have a physical inability to behaviourally thermoregulate and cannot express their thermal preference. Because of their inability to use microclimatic variations, Gleason *et al.* (2023) posited that embryonic brown anole lizards, *Anolis sagrei,* would have enhanced physiological thermal tolerance flexibility. In this way, embryonic lizards circumvent ‘The Bogert Effect’, which predicts that the ability of organisms to behaviourally thermoregulate, and thereby dampen temperature extremes, constrains the evolution of thermal tolerance plasticity (Bogert, 1949; Muñoz and Bodensteiner, 2019). Gleason *et al.* (2023) found that heat-hardened embryos had greater heat tolerance, but the hardening process itself increased embryo heart rate, which was indicative of the energetic cost to hardening. Nevertheless, their data are consistent with lizard embryos having the capacity for adaptive plasticity when faced with warming temperatures, which is predicted to be limited in adult lizards by ‘The Bogert Effect’. This example illustrates the importance of considering developmental stage when making predictions of tolerance and survival. As early life stages are often the most sensitive when it comes to environmental perturbation, considering the extent to which a dynamic physiological/behavioural relationship (in this case constraints in behaviour, over development) contributes to changes in sensitivity will allow for better survival and tolerance predictions, ultimately improving conservation and management practices.

III. In life stages that can behaviourally thermoregulate, the capacity for, or costs of, physiological acclimation may be obscured, making the interplay between behaviour and physiology seem counterintuitive. When animals can use thermal refugia, such as microclimates, behavioural choices may not always align with ‘physiological optima’ as predicted in traditional studies of thermal physiology. For example, Salachan *et al.* (2021) showed that *Drosophila* responses to changing environmental conditions were different depending on whether individuals were temperature restricted or allowed a choice of temperature. When individual fruit flies were temperature restricted and only physiological capacity was considered, higher temperatures led to increased reproductive performance within a range of certain permissible temperatures. Following a typical thermal performance curve, egg production increased with temperature until it decreased at the highest temperature tested. Furthermore, egg-laying capacity increased in fruit flies acclimated to a higher temperature (i.e. context), likely linked to physiological processes such as higher metabolic rates at the higher temperatures. On the other hand, when individuals were allowed a choice of temperature in which to lay their eggs, fruit flies buffered against increasing temperatures by leveraging microclimate variation, regardless of thermal experience (i.e. even if they were acclimated to a higher temperature). This example provides a case for the importance of a nuanced understanding of both physiology and unconstrained behaviour (i.e. allowing the choice of environment) to make predictions of how organisms may survive an environmental challenge. In this case, despite having the physiological capability to survive with enhanced reproduction in a warmer environment, if permitted to move, an organism might choose otherwise. It is possible that this behavioural thermal preference is driven by other physiological needs (see *Example IV* below). For real-world relevance, an understanding of how habitats and landscapes may arrange to create microclimates would further our appreciation of how organisms choose to use them.

IV. Similarly, behavioural preference may not always follow what might be predicted through evolutionary adaptation to environmental change. Pilakouta *et al.* (2023b) challenged the assumption that physiological adaptation to elevated temperatures over successive generations would alter behavioural temperature preference. Exploiting natural habitats in Iceland, the group compared populations of the freshwater three-spined stickleback (*Gasterosteus aculeatus*) living in lakes warmed by geothermal activity to sticklebacks living nearby in ambient-temperature lakes. Previous studies had shown physiological and morphological differences between these populations of stickleback, for example, sticklebacks from warm lakes had a lower standard metabolic rate, a deeper body and a shorter jaw (Pilakouta *et al.*, 2020, 2023a). Based on the finding that standard metabolic rate and temperature preference have been shown to be negatively correlated (Killen, 2014), the group predicted that fish from warm habitats would prefer higher water temperatures. In contrast to this prediction, they found that temperature preference was not habitat based. Even though the warm-adapted fish clearly persisted in the higher temperature lakes and have been exposed to warm temperatures for hundreds to thousands of years, they still preferred the same lower temperature (13°C) as fish from the colder lakes illustrating that adaptive potential based on physiology are not always predicted by behavioural thermal preference. In this case, both warm- and cold-adapted Icelandic stickleback populations had improved acquired immunity at 13°C (Franke *et al.*, 2017, 2019), demonstrating an underlying physiological need that is a greater influence on thermal preference than physiological capability to tolerate the heat. Like egg laying in fruit flies, in stickleback, physiological mechanisms of temperature tolerance and behavioural thermal preference are misaligned, emphasizing complexities that need to be considered when setting conservation policy.

**Figure 2:**
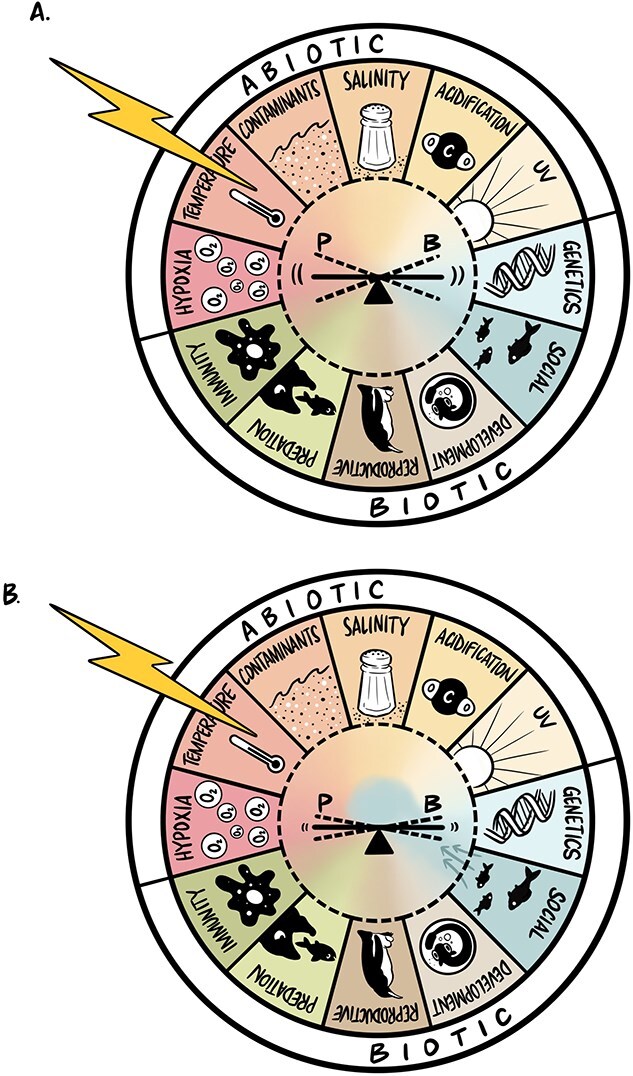
In this example, panel (**B**) represents an individual experiencing a greater influence from social factors than the individual in panel **A**. When exposed to the same external temperature challenge (in this case, high temperature), represented by a lightning bolt, the context of the individual shapes the way the external challenge interacts at the physiology/behaviour interface.

V. Many animals exist in social groups at some point in their life history. Sociality and social living can reduce chances of predation (Ioannou, 2017), increase foraging success (Harpaz and Schneidman, 2020) and provide energetic savings (Marras *et al.*, 2015; Zhang and Lauder, 2023). An animal’s social context will therefore interact with the physiology/behaviour interface and can influence the way an animal responds to an environmental challenge. Melanson *et al.* (2023) demonstrated that a short period of social experience (i.e. 24 h) altered thermal sensitivity in that fish voluntarily emerged from water at a higher temperature than fish who were socially naïve (Fig. 2). Here, a relatively short-term change in social context affected the subsequent response to an environmental challenge (Fig. 2). The change in thermal sensitivity with social interactions appears to be at least partly explained by a putative change in sensitivity of a highly conserved membrane ion channel, considered a key physiological temperature regulator, transient receptor potential vanilloid 1 (TRPV1). Notably, acute aggressive dyadic contests also elicited these TRPV1-mediated changes in thermal behaviour (Currie *et al.*, unpublished). Here, a change in context (e.g. social interactions), leads to changes in both physiology and behaviour, affecting how fish sense temperature, potentially altering thermal susceptibility (Currie and York, 2026). As also pointed out by Killen *et al.* (2026), this example emphasizes how understanding social context is imperative for understanding ecological sensitivities with implications for species’ conservation and management best practices.

VI. Behavioural responses to an environmental challenge can also be affected when reproductive factors shape an individual’s context. This idea was illustrated recently by Noiret *et al.* (2026) who looked at thermoregulatory behaviour in king penguins (*Aptenodytes patagonicus*) in response to the environmental challenge of heat. The group found that panting was the most observed thermoregulatory behaviour, followed by wing spreading, and finally, exposing the brood patch—a highly vascularized area used to protect the egg/chick but also potentially to dissipate heat. The distribution of these behavioural responses was affected to a certain extent by sex and to a greater extent by reproductive status. King penguins, keeping the hatched chick warm (brooding), generally present more signs of heat stress than penguins incubating the egg, having higher subcutaneous body temperatures despite an increase in the frequency of wing spreading and enhanced brood patch exposure. In addition, in exposing the brood patch more frequently, a chick is more susceptible to predation risk than the egg. These data show that king penguin thermoregulatory behaviour varies with reproductive status, which then has implications on reproductive success. Brooding penguins with young at the chick stage show a higher susceptibility than incubating penguins with young at the egg stage. There are likely costs associated with brooding penguins living with a higher body temperature, for example, increased need to feed or higher susceptibility to other environmental stressors. At the level of conservation and management, understanding the trade-offs, costs and differential sensitivities that may be associated with reproductive status is critical to gaining a complete understanding of how to protect these species.

## Conclusion

In these few examples, we illustrate how studying animals in their environment in a more holistic way, accounting for how abiotic and biotic influences both shape an animal’s context and act as discrete environmental challenges, is critical for addressing the conservation and biodiversity implications of rapid environmental change. While the limited view that physiology or behaviour is the most sensitive indicator of environmental change is diminishing, widespread acceptance of the need to address existing complexities has not yet occurred. Our examples above contribute to our understanding of animal limits, vulnerabilities and adaptation potential and strengthen our understanding of the context-dependent nature of an animal’s response to environmental change. As experimental biologists, we are limited in the complexities we can represent in a laboratory situation and the physiological and behavioural variables we choose to measure. Although it may seem straightforward to avoid predicting the effects of one mechanistically distinct process on another, careful consideration of mechanisms of action—drawing on frameworks such as Adverse Outcome Pathways (Ankley *et al.*, 2010)—is essential when designing experiments and validating links between physiological and behavioural traits for conservation and management applications. Mesocosm and field studies certainly enhance our understanding further but still tend to capture only a fraction of the complexities that shape the physiological/behavioural interface.

Within ecotoxicology (Ankley *et al.*, 2010) and multiple stressor research (Simmons *et al.*, 2021), the need to capture the complexity of interactions through statistical modelling is clearly recognized. Rather than continue to generate separate sets of empirical data on these interactions across a small context range, our priority as experimental biologists must be to work with statistical modellers to simulate the complexity of the organisms we work with and generate empirical data to fill identifiable gaps in our knowledge. Modelling these complex interactions will allow us to identify priorities for conservation science, for example, understudied external environmental challenges and physiology–behaviour relationships that require further interrogation. Identified conservation challenges can then be addressed through simulation modelling, allowing a greater and more nuanced understanding of predicted outcomes. The increasing need to inform conservation measures through an integrated approach must be matched by our ability to work across many disciplines, including the alignment with frameworks that go beyond the individual and encompass a whole system approach (Simmons *et al.*, 2021). In a recent perspective on how physiology can be integrated into the conservation process, Seebacher *et al.* (2023) emphasized the unique role physiology plays at the interface between environment and organisms. We agree and consider that any environmental change and/or challenge will first affect physiology *and* behaviour, with physiology influencing behaviour or *vice versa*. Early identification of the challenges faced by individuals/population is often the first step in the conservation process. Thus, considering both physiological and behavioural dependent variables within the full internal and external context of organisms will lead to more inclusive biological assessments and robust conservation decisions and interventions.
